# Topical anesthesia in phacoemulsification

**DOI:** 10.4103/0974-620X.71892

**Published:** 2010

**Authors:** Saad Waheeb

**Affiliations:** Department of Ophthalmology, King Abdulaziz University, College of Medicine, Jeddah, Saudi Arabia

**Keywords:** Clear cornea phacoemulsification, patient and surgeon satisfaction, sedation, topical anesthesia

## Abstract

**Purpose:**

To evaluate the efficacy of topical anesthesia; topical Benoxinate 0.4% (Oxybuprocaine) and Xylocaine (Lidocaine) gel, in selected cataract patients as an alternative to peribulbar or retrobulbar block anesthesia during cataract surgery.

**Materials and Methods:**

Prospective non-comparative evaluation of patients’ and surgeon’s satisfaction at the end of the procedure. Three hundred patients (300 eyes) were included in the study. The procedure was explained to patients with details regarding what will happen and what to expect during surgery. All patients received topical anesthesia with Benoxinate 0.4% eye drops and Xylocaine gel 2%. All surgeries were done by the same surgeon using the same machine (updated LEGACY phacoemulsifier, Alcon) and approach (clear corneal incision) and followed by a foldable intraocular lens (IOL) implantation.

**Results:**

None of the patients had severe pain during the procedure; only 2% (six of 300) required use of intravenous sedation (Propofol), both the surgeon’s and the patients’ satisfaction were high. Eye movements and blepharospasm were not significant problems, and no serious complications occurred. Rate of vitreous loss due to posterior capsule tear/rupture was within literature reported range and not different from our previous experience.

**Conclusion:**

Topical anesthesia is a satisfactory and safe alternative to retrobulbar and peribulbar anesthesia for clear corneal phacoemulsification and intraocular lens implantation in selected cataract patients in the hands of experienced cataract surgeon.

## Introduction

Cataract surgery is the most commonly performed surgery in ophthalmology. The surgery has gone through major evolutions throughout its history, from ‘couching’ to intracapsular, extracapsular, and nowadays phacoemulsification. The search for a safe and effective technique for anesthesia has also been evolving. Some surgeons have been operating the patient under general anesthesia (G/A). Although it is convenient for the surgeon, it subjects the patient to all the complications of G/A. The retrobulbar block anesthesia method supplemented by facial nerve block (Atkinson, O’Brien, Van Lint, etc) was the gold standard for many decades, but had the disadvantages of being a “blind injection” with the potential of causing perforation, hematomas, or intrathecal/ central nervous system spread.[1–6] Peribulbar injections avoided the need for facial block, and minimized the incidence of optic nerve damage, but again was a blind procedure with two injections usually needed.[7,8]

Topical anesthesia has the advantage of avoiding all the problems associated with the above injections. The aim of our study has been to evaluate the efficacy and safety of topical anesthesia in clear corneal phacoemulsification and intraocular lens implantation in the Saudi population in terms of patients’ and surgeon’s satisfaction and rate of complications and rate of significant blepharospasm intraoperatively.

## Materials and Methods

This is a prospective non-comparative study conducted at the King Faisal Specialist Hospital and Research Centre -Jeddah branch between March 2007 and 2009. The study included 300 patients (300 eyes) whom underwent phacoemulsification under topical anesthesia by the same surgeon (S. W). All patients were evaluated in the clinic for refraction, anterior segment examination, applanation tonometry, and dilated indirect fundus examination using the full illumination of the instrument. Biometry was done, routine blood tests and electrocardiogram (ECG) requested, and patients were sent for anesthesia clearance regarding the possibility and safety of intravenous (IV) sedation administration.

Certain groups of patients were determined as “unfit” for topical anesthesia and these were not included in the study. Those were patients who had communication problems (short of hearing, language barriers, dementia, etc.). Patients who were not cooperative during applanation tonometry or who were excessively photophobic during indirect fundus examination were also excluded. Patients who had very small and difficult to dilate pupils with the anticipated use of iris dilators or sphincterotomies, and pseudoexfoliation (PXS) were excluded. Brown cataracts or anticipated long surgeries (over 20 min) were also not included for topical anesthesia.

All selected patients were counseled regarding the steps of surgery, and what they should expect during it. They were told they will need to be relaxed, avoid closing their eyes, and avoid excessive motility. All patients signed an informed consent.

On the day of surgery, all patients were brought on fasting, Benoxinate 0.4% eye drops were instilled twice, 5 min apart, then 2% Xylocaine gel. Gel was put in the conjunctival sac 10 min before draping. Additional Benoxinate 0.4% eye drops (preservative-free minims) were available for use intra-operatively if needed. Patients who expressed severe discomfort or who were excessively anxious during surgery were administered low grade sedation (Propofol infusion).

All surgeries were performed by the same surgeon using clear cornea 2.2-3.0 mm incision, wide anterior continuous curvilinear capsulorrhexis (ACCC), hydrodissection, phacoemulsification using the Everest (updated legacy-Alcon) or Infinity machines, and automated irrigation/aspiration (I/A). A foldable posterior chamber intraocular lens (PCIOL) was injected using the manufacturer’s recommended injector.

Post-operatively, the level of patient’s pain and / or discomfort was recorded by the operating room (OR) staff nurse as mild, moderate, or severe. The surgeon’s satisfaction was reported by the assistant surgeon attending surgery and was assessed by the degree of patient’s cooperation, eye motility, squeezing of the eyelids, excessive positive pressure, and rate of complications.

## Results

**Figure 1:**
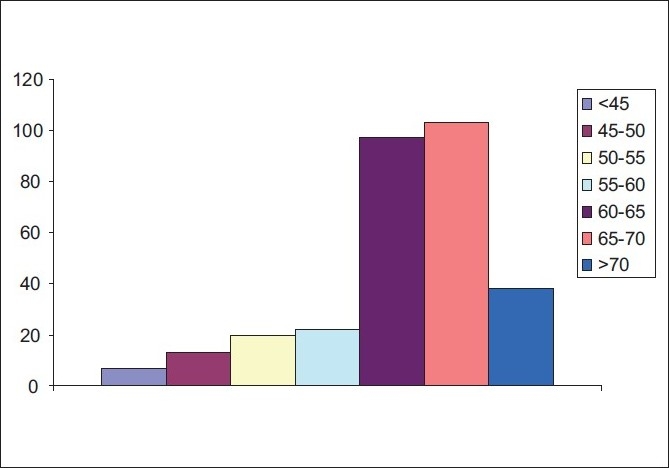
Patients’ age distribution

Table 1 and Figure 1 show patients’ gender and age distribution, respectively. Mean age of the study patients was 65 years. Most of the patients (228/300; 76%) were on anticoagulants (Aspirin and/or Plavix), for systemic vascular disease. These were stopped few days to a week before the surgery. All patients were cleared by a pre anesthesia check up regarding the possible use of low grade intravenous sedation during surgery. Average surgery time was 20 min +/- 5 min (measured from the corneal incision to the removal of the speculum). Corneal incision was sutured (10/0 vicryl) in 150 patients (50%).

**Table 1:** Patients’ gender distribution

*Males*	*Females*	*Total*
160	140	300

Vitreous loss due to posterior capsule (PC) rent or zonular dialysis was encountered in three eyes of three patients (1.0% of the cases). This rate is well within the reported complication rate of experienced surgeons in literature.[9–11] All cases had the PCIOL successfully implanted either in the capsular bag or in the sulcus area.

**Figure 2:**
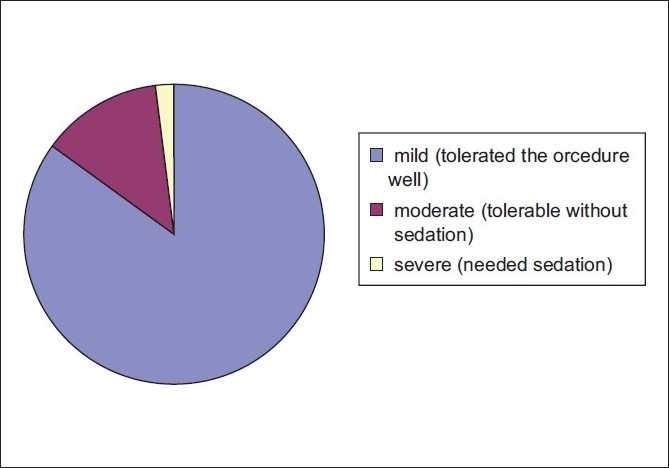
Level of patient’s pain and/or discomfort

A total of 255 out of 300 (85%) patients tolerated the procedure well with no need for intravenous sedation, while 39 of 300 (13 %) reported moderate discomfort yet tolerable without sedation. Only 2% of patients reported severe pain, discomfort or anxiety that needed sedation [Figure 2].

**Figure 3:**
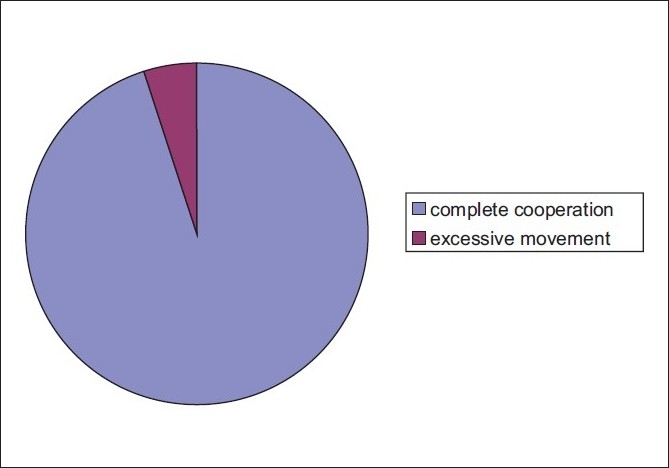
Intraoperative patients’ behavior/cooperation

The surgeon was very comfortable with 285 of 300 (95%) patients [Figure 3], with good patient cooperation and no adverse outcomes. Only 15 patients (5%) had excessive eye motility that was usually well controlled by talking to the patient, assuring and instructing him/her. We did not encounter major complications related to eye motility or positive pressure.

**Figure 4:**
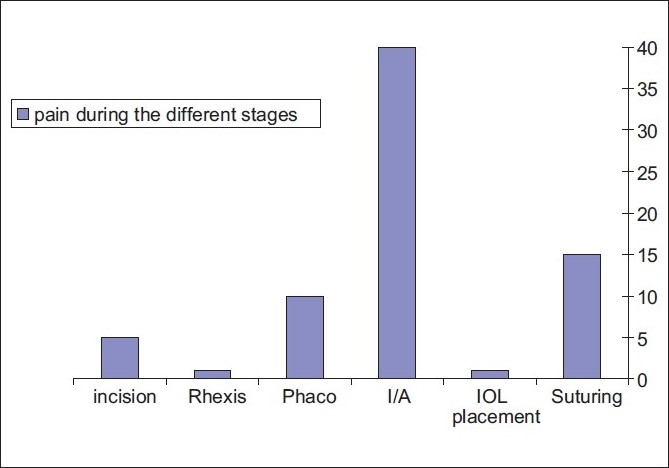
Number of patients with significant pain during different surgery steps

Most of the pain and complaints from the patients were during the introduction of the irrigating fluid to the eye with the sudden deepening of the AC [Figure 4]. The incision, rhexis, hydrodissection, phacoemulsification, and IOL insertion were all well tolerated. Some discomfort or pricking sensation was felt when a suture was applied, but that resolved with the addition of topical Benoxinate 0.4% drops. Patients who were most distressed were those with prolonged surgeries, and persons who are anxious by nature.

Overall, surgeon satisfaction was 100%; even cases that were not cooperative were well managed after sedation. A total of 255 patients (85% of patients) were satisfied with the anesthesia during the surgery and did not need any sedation. Those who needed sedation were also satisfied postoperatively due to the amnesia caused by the Propofol and were willing to be operated on the fellow eye under topical anesthesia.

## Discussion

The first documented treatment of cataract is couching where its history dates back to about the fifth century BC in India.[12] The procedure involved displacing the lens from the pupil into the vitreous cavity. Patients could see better, but vision was still blurry because no corrective lenses were available at that time. In 1747, Jacques Daviel, MD, after performing a failed couching procedure, completed the first cataract extraction by removing a patient’s lens from its normal position behind the iris. Cataract surgery is still performed today using a similar form of this technique.

The first use of topical anesthesia in modern times was by Koller in 1884 that used Cocaine. In general, ocular anesthetics belong to one of two groups, either ester or amide. The ester group includes Oxybuprocaine 0.4% (Benoxinate), which is the most commonly used due to its high degree of safety. Tetracaine 0.5 or 1.0% and Proparcaine also belong to the same group with a short duration of action (20 min) but are the least toxic to the epithelium.[13]

Lidocaine 4% (Lignocaine) and Bupivacaine 0.5% and 0.75% both belong to the amide group. They have a longer duration of action but are associated with an increased corneal toxicity.

Our experience with topical anesthesia has been very encouraging. In fact we have been using it for almost 95% of our patients for the past three years. Our results are in line with the results of Monestam *et al*, who tried topical anesthesia in an unselected population of 890 patients in Sweden, operated upon by four surgeons, in which 72% of patients were operated successfully without the need for any sedation. Of the 890 patients, only one chose not to repeat the procedure under topical anesthesia.[14]

The question of which topical anesthetic to use was the subject of a lot of research. Martini *et al*, compared topical *Ropivacaine 1%* versus *Lidocaine 4%* regarding their anesthetic effect and effect on endothelial cell count.[15] They concluded that Ropivacaine was as effective as Lidocaine 4% in terms of anesthesia and safety. *Tetracaine* was also compared to *Lidocaine 2%* gel in a study in Wilmer eye institute and it was found that a single application of Lidocaine gel is similar to multiple applications of Tetracaine eye drops in achieving anesthesia and both caused no significant toxicity to the ocular surface.[16]

If the patient is found to be anxious and the anesthesia inadequate from topical anesthesia, a simple option would be to use intracameral preservative-free Lidocaine. Elvira *et al*, used topical anesthesia plus 1% Lidocaine 0.3 ml intracameral in 19 patients and he evaluated the endothelial cell count preoperatively and at one and three months postoperatively.[17] Lidocaine 1% intracameral was a safe supplementation to topical anesthesia in phacoemulsification.

But does the surgeon factor have a role in the success of surgery using topical anesthesia? Speed in performing surgery is a factor that can minimize patient’s discomfort and anxiety. Fluctuations in the anterior chamber depth as in wound leak or excessive instrument traffic through the wounds will cause more discomfort to the patient. In fact, Mathew *et al*, compared patient’s pain level and discomfort when operated upon by an experienced surgeon, versus a surgeon who was still in the learning curve.[18] He reported that topical anesthesia with Proparacaine provided similar and reasonable anesthetic effects in patients having surgery by a surgeon in the learning curve and those having surgery by an experienced surgeon. Although the discomfort perceived during surgery performed by an experienced surgeon was less, it was not statistically significantly different.

Vitreous loss complication rate was comparable to and well within that reported in the literature, of 0.8-1.25%.[9–11]

## Conclusion

Topical anesthesia was proved to be an effective and safe option in cornea-based phacoemulsification and intraocular lens implantation in selected patients, in our experience. However, the success depends to a great extent on surgeon’s preference and experience; and proper patient’s selection, counseling, and preparation. It is a safe alternative to the use of retro- or peribulbar injections, less time consuming and definitely less costly which has a measureable impact in high-volume settings and on the national level.
